# Myocarditis causing severe heart failure - an unusual early manifestation of leptospirosis: a case report

**DOI:** 10.1186/s13104-015-1031-1

**Published:** 2015-03-13

**Authors:** Jagath Pushpakumara, Thushanthy Prasath, Ganaja Samarajiwa, Sugandika Priyadarshani, Nilanka Perera, Jegarajah Indrakumar

**Affiliations:** Ward 01, Colombo South Teaching Hospital, Kalubowila, Sri Lanka; Department of Medicine, Faculty of Medical Sciences, University of Sri Jayewardenepura, Kalubowila, Sri Lanka; University Medical Unit, CSTH, Kalubowila, Sri Lanka; Department of Medicine, Faculty of Medical Sciences, USJP, Nugegoda, Sri Lanka

**Keywords:** Leptospirosis, Myocarditis, Left ventricular dysfunction

## Abstract

**Background:**

Leptospirosis is the most widespread zoonosis in the world. Cardiac involvement is a frequent complication of leptospirosis although significant left ventricular dysfunction is rare. We report a case of fatal leptospira myocarditis leading to cardiogenic shock on the second day of illness. This early occurrence of myocarditis is not previously reported.

**Case presentation:**

A 36-yr-old previously healthy Sri Lankan male who takes care of a horse presented to the medical casualty ward with a one day history of fever, arthralgia and severe myalgia. He developed hypotension on the second day of illness. Electrocardiogram showed sinus tachycardia with ST segment depression in lateral leads which evolved in to rapid atrial fibrillation in the subsequent days. 2D echocardiogram showed dilated cardiac chambers with severe global hypokinesia and an ejection fraction of 20%. His renal and liver functions were within normal limits. He developed multi organ dysfunction syndrome and refractory shock, later in the course of illness.

Leptospirosis was confirmed by positive leptospira IgM and negative IgG. Patient died on the fifth day of illness despite optimal medical treatment with intravenous penicillin, meropenem, levofloxacin, inotropes and supportive care in the intensive care unit.

**Conclusions:**

We describe a rare and unusual early complication of leptospirosis which has not been reported before. It is important to bear in mind that leptospirosis could present as myocarditis during the early phase of illness.

## Background

Leptospirosis is an infectious disease of animals and humans caused by the pathogenic spirochetes of the genus *Leptospira*. It is the most widespread zoonosis in the world [1] and is re-emerging globally [2]. Leptospirosis is found commonly in the tropical countries and Sri Lanka reports an annual incidence rate of 31–164 per 100,000 populations [3]. Anicteric leptospirosis resembles a simple febrile illness causing diagnostic difficulty. The mortality in leptospirosis is mainly due to more serious manifestations such as myocarditis, acute renal failure, hepatitis, pulmonary hemorrhage and multi-organ failure.

Leptospirosis is a biphasic illness characterized by an early septicaemic phase lasting about a week and a delayed immune phase [1]. Most complications occur during the immune phase which has raised the possible pathogenic mechanisms described up to now. However, pathophysiology of cardiac involvement in leptospirosis is poorly understood [4]. Most studies reveal that cardiac involvement in the form of ECG (electrocardiogram) abnormalities or trans thoracic echo abnormalities are frequent and probably underestimated [1,3,5,6]. Autopsy studies also report a significant cardiac involvement in fatal leptospirosis [7]. But data reveal that severe cardiac dysfunction in these patients are rare [3,6,8] and all published literature report the occurrence of myocarditis beyond the first 5 days – 1 week of illness [5,9,10].

We report a case of fatal leptospira myocarditis leading to cardiogenic shock on the second day of illness. Patient rapidly deteriorated due to severe myocarditis leading to refractory shock and passed away on the fifth day of illness despite optimal medical care. This case is unusual for the development of early myocarditis in anicteric leptospirosis.

## Case presentation

A 36-yr-old previously healthy Sri Lankan male who takes care of a horse presented to the medical casualty ward with fever, arthralgia and myalgia for one day. He complained of mild dysuria but had normal urine output. He did not have chest pain or shortness of breath. Further inquiry revealed that he was treated for leptospirosis during a febrile illness in the past. On examination, patient was afebrile, anicteric. His blood pressure was 90/60 mmHg and pulse rate 76 bpm. Rest of the examination was unremarkable. Initial investigations performed on the day of admission revealed neutrophil leukocytosis, mild thrombocytopaenia and microscopic haematuria (Table 1). Renal function and liver enzymes were within normal limits. Urine analysis revealed pus cells 05 – 06 /hpf, red cells 30 – 35 /hpf. CRP (C-reactive protein) was 75 mg/dl. A clinical diagnosis of leptospirosis was made and he was started on intravenous penicillin in addition to adequate hydration.

**Table 1:** **Demonstrating the serial investigations**

**FBC**	**D**_**1**_	**D**_**2**_	**D**_**3**_	**D**_**4**_	**D**_**5**_
WBC	10.5 × 10^3^/mic L	10.5 × 10^3^/mic L	1.6 × 10^3^/mic L	3.5 × 10^3^/micL	3.1 × 10^3^/micL
N	95%	91%	59%	62%	58%
L	4%	5.7%	32%	36%	38%
Hb	12.9 g/dl	11.3 g/dl	9.1 g/dl	9.8 g/dl	10.1 g/dl
PCV	40%	33%	28%	32%	34%
Platelet	102 × 10^3^/mic L	55 ×10^3^/mic L	10 × 10^3^/mic L	46 × 10^3^/micL	36 × 10^3^/micL
**S. Creatinine**	70 micmol/L	103 micmol/L	234 micmol/L	269 micmol/L	362 micmol/L
**ALT**		48 IU/L	80 IU/L	188 IU/L	2726 IU/L
**AST**		42 IU/L	197 IU/L	235 IU/L	7438 IU/L

**Figure 1:**
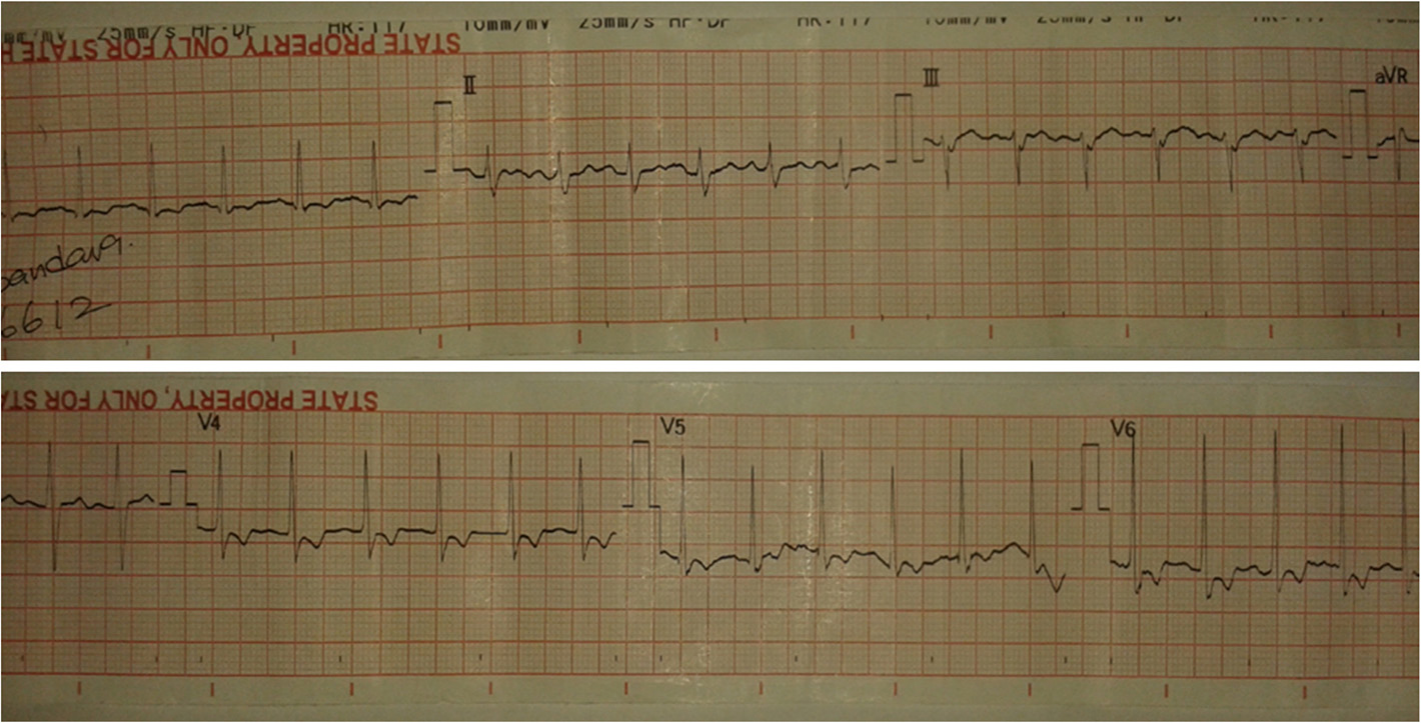
**ECG ,demonstrating sinus tachycardia, ST depressions and T inversions in leads V**_**4**_ **– V**_**6**_ **on 2nd day of illness.**

**Figure 2:**
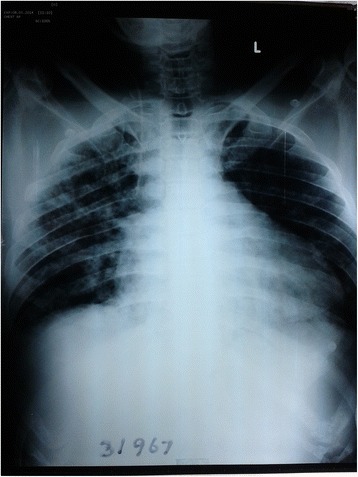
**CXR showed gross cardiomegaly and bilateral pulmonary shadows on the 3rd day of illness.**

**Figure 3:**
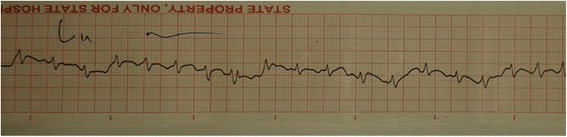
**ECG showing rapid atrial fibrillation, developed on the 3rd day.**

On the second day of illness, he developed hypotension with tachycardia and dyspnoea. ECG revealed sinus tachycardia with ST depression in leads V_4_ – V_6_ [Figure 1]. His CXR (chest x ray) showed gross cardiomegaly and bilateral pulmonary shadows [Figure 2]. CVP (central venous pressure) was 16 cmH_2_O and 2D echocardiogram revealed dilatation of all four chambers, severe global hypokinesia and ejection fraction of 20%. Troponin I was 12.77 ng/ml (normal range < 0.40). He was diagnosed to have early and severe myocarditis. Patient was transferred to ICU (intensive care unit) as he needed inotropic support. Intravenous antibiotics including penicillin, meropenem and levofloxacin were continued. Patient was electively ventilated on the 3rd day of illness due to severe respiratory distress. On the same day, he developed rapid atrial fibrillation [Figure 3] unresponsive to digoxin and intravenous amiodarone. Platelet count dropped to 10 × 10^3^ μ/L and he was given platelet transfusions to prevent bleeding [Table 1]. We started intravenous methylprednisolone based on previous studies showing a mortality benefit in severe leptospirosis complicated with myocarditis [11]. His serum creatinine increased to 362 μ mol/L by the 5th day of illness and ALT (alanine transaminase) and AST (aspartate transaminase) were markedly elevated up to 2726 U/l and 7438 U/l respectively. Dengue and Mycoplasma infections were excluded. Blood cultures were negative. Leptospira IgM antibody performed on the 5th day of illness was positive (IgG negative). Patient developed refractory shock and died of persistent ventricular tachycardia despite optimal treatment in the ICU.

## Discussion

Leptospirosis is a life threatening disease that can present as a mild anicteric illness (90% of cases) or as a severe icteric disease [6]. Our patient presented with early myocarditis leading to severe left ventricular dysfunction. Myocarditis in this patient was characterized by persistent hypotension, tachycardia leading to atrial fibrillation, ST segment depression in ECG, elevated cardiac markers and dilated cardiac chambers with LV (left ventricular) dysfunction. Apart from myocarditis, leptospirosis can be complicated with pulmonary hemorrhage, hepatitis causing fulminant hepatic failure, acute kidney injury and ocular manifestations. However, most patients with leptospirosis recover completely without permanent residual effects [5,9].

Previous studies reveal that cardiac involvement is common in leptospirosis [3,6,8]. A study conducted in India found that 56% of leptospirosis patients had cardiac involvement and 52% of them had ECG abnormalities [6]. Commonest ECG changes were conduction defects followed by ST/T wave changes and atrial arrhythmias [3,6,8]. But significant LV dysfunction was not seen. A study conducted in Sri Lanka during the 2011 outbreak found 15.6% of patients to have myocarditis [3]. The degree of LV dysfunction was not documented. However, there were no fatalities. Prevalence of myocarditis in leptospirosis increased from 10.3% in 2008 to 15.6% in 2011 according to this study. This data reveal that despite the more frequent occurrence of myocarditis in leptospirosis, severe LV dysfunction and mortality is still rare.

Pathophysiology of cardiac involvement in leptospirosis is largely unknown. Autopsy studies show an interstitial myocarditis with infiltration of predominantly lymphocytes and plasma cells, petechial hemorrhages (particularly in the epicardium), mononuclear infiltration in the epicardium, pericardial effusions, and coronary arteritis [1] vasculitis was proposed as the principal pathogenic mechanism [8]. Recent literature reports that activation of Toll-like receptor (TLR) 2 is responsible for renal and pulmonary manifestations in leptospirosis [9]. This immunological basis explains the development of myocarditis after the initial one week of illness. There are no reported cases of leptospirosis causing myocarditis presenting as early as day two of illness.

Our patient probably developed an unusually early immune phase as evidenced by early and severe myocarditis on the second day of illness. It is possible that immune reaction may have been triggered early by the presence of low levels of preformed antibodies resulting from the first episode of leptospirosis which occurred 15 years before.

This case illustrates that in endemic areas of leptospirosis, a possible second episode of this common illness may cause early and severe manifestations leading to diagnostic difficulty and high mortality.

## Conclusion

Myocarditis in leptospirosis can rarely lead to fatal cardiac dysfunction and could manifest during the early part of anicteric leptospirosis despite normal renal, hepatic functions. A high degree of suspicion is needed to diagnose and treat this life threatening complication early.

## Consent

Written informed consent was obtained from the patient’s next of kin for publication of this Case Report and any accompanying images. A copy of the written consent is available for review by the Editor-in-Chief of this journal.

## Abbreviations

LV: Left Ventricle
ECG: Electrocardiogram
CXR: Chest X ray
CRP: C reactive protein
CVP: Central Venous Pressure
ICU: Intensive Care Unit
TLR: Toll like Receptors
ALT: Alanine Transaminase
AST: Aspartate Transaminase
